# Gut microbiota-derived hexa-acylated lipopolysaccharides enhance cancer immunotherapy responses

**DOI:** 10.1038/s41564-025-01930-y

**Published:** 2025-02-10

**Authors:** Puspendu Sardar, Benjamin S. Beresford-Jones, Wangmingyu Xia, Omar Shabana, Satoshi Suyama, Ruben J. F. Ramos, Amelia T. Soderholm, Panagiotis Tourlomousis, Paula Kuo, Alexander C. Evans, Charlotte J. Imianowski, Alberto G. Conti, Alexander J. Wesolowski, Natalie M. Baker, Emily A. L. McCord, Klaus Okkenhaug, Sarah K. Whiteside, Rahul Roychoudhuri, Clare E. Bryant, Justin R. Cross, Virginia A. Pedicord

**Affiliations:** 1 Cambridge Institute of Therapeutic Immunology and Infectious Disease, University of Cambridge, Cambridge, UK; 2 Department of Medicine, School of Clinical Medicine, University of Cambridge, Cambridge, UK; 3 Donald B. and Catherine C. Marron Cancer Metabolism Center, Memorial Sloan Kettering Cancer Center, New York, NY USA; 4 Department of Veterinary Medicine, School of Biological Sciences, University of Cambridge, Cambridge, UK; 5 Department of Pathology, School of Biological Sciences, University of Cambridge, Cambridge, UK

**Keywords:** Immunotherapy, Microbiome, Cancer

## Abstract

The gut microbiome modulates immunotherapy treatment responses, and this may explain why immune checkpoint inhibitors, such as anti-PD-1, are only effective in some patients. Previous studies correlated lipopolysaccharide (LPS)-producing gut microbes with poorer prognosis; however, LPS from diverse bacterial species can range from immunostimulatory to inhibitory. Here, by functionally analysing faecal metagenomes from 112 patients with melanoma, we found that a subset of LPS-producing bacteria encoding immunostimulatory hexa-acylated LPS was enriched in microbiomes of clinical responders. In an implanted tumour mouse model of anti-PD-1 treatment, microbiota-derived hexa-acylated LPS was required for effective anti-tumour immune responses, and LPS-binding antibiotics and a small-molecule TLR4 antagonist abolished anti-PD-1 efficacy. Conversely, oral administration of hexa-acylated LPS to mice significantly augmented anti-PD-1-mediated anti-tumour immunity. Penta-acylated LPS did not improve anti-PD-1 efficacy in vivo and inhibited hexa-acylated LPS-induced immune activation in vitro. Microbiome hexa-acylated LPS therefore represents an accessible predictor and potential enhancer of immunotherapy responses.

## Abstract (web summary)

Hexa-acylated lipopolysaccharides from the gut microbiota are associated with efficacy in patients with cancer receiving anti-PD-1 and stimulate host immune activation for enhanced anti-tumour immunity in mouse models.

## Main

Immune checkpoint inhibitors (ICI) unleash the power of a patient’s own immune system to combat cancer. Multiple landmark studies have identified a role for the gut microbiome in modifying ICI treatment response^1,2^; however, there is little consensus between these studies as to which members of the gut microbiota are important for modifying treatment outcomes. More recent studies instead conclude that the relationship between the gut microbiome and ICI clinical response extends beyond the level of species composition^3^, pointing to associations between unfavourable immunotherapy outcomes and lipopolysaccharide (LPS)-producing bacteria in the gut microbiota^4^. However, beyond association, the effects of microbiota-derived LPS remain incompletely defined, and few studies consider the different structures of LPS produced by gut microbes.

Primarily characterized in pathogenic infections for its role in stimulating immune responses to Gram-negative bacteria, LPS activation of host toll-like receptor 4 (TLR4) has complex context-dependent roles in physiology. In the gut, LPS-TLR4 signalling has been shown to both dampen intestinal immune responses to the resident microbiota during states of health^5^ and modulate systemic autoimmune activity^6^. However, the immunostimulatory effect of LPS depends on its structure, which differs profoundly between bacterial species. Indeed, not all Gram-negative bacteria in the gut harbour all genes within the LPS biosynthetic pathway, resulting in production of a heterogenous pool of LPS structures by different gut microbes. The lipid A region of LPS that is recognized by TLR4 is composed of two glucosamine sugars linked to a variable number of acyl chains. In humans, hexa-acylated LPS potently activates TLR4 and host immunity, whereas hypo-acylated penta- and tetra-acylated LPS poorly activate TLR4 and can antagonize immune activation by hexa-acylated LPS^6–9^. Recent studies have associated some LPS-encoding Gram-negative taxa, such as members of the *Bacteroidaceae* family, with non-response to ICI treatment^4,10,11^. However, taxonomic associations with treatment response may not capture the diversity of LPS structures present and the resulting complexity of functional interactions occurring between gut-derived LPS species and host immune responses.

To determine the potential functional rather than taxonomic basis for Gram-negative bacteria-mediated modification of ICI response, we analysed LPS biosynthesis genes in faecal metagenomes of patients with cancer undergoing ICI with anti-PD-1 therapy. Using a multi-cohort meta-analysis combining taxonomic and functional annotation, we identify hexa-acylated LPS-encoding gut bacteria as a potential determinant of clinical response to anti-PD-1 therapy. Using both human and mouse in vitro assays and an in vivo mouse tumour model, we demonstrate a TLR4-dependent causative role for increased ratios of hexa-acylated LPS in the gut in enhancement of anti-PD-1-mediated anti-tumour responses. These findings further our understanding of the complex host–microbiome interactions that define response to ICI treatment and advise against the use of inhibitors of LPS-induced TLR4 signalling suggested by some previous studies^12,13^.

## Results

### Patient microbiome LPS segregates responders to anti-PD-1

**Fig. 1:**
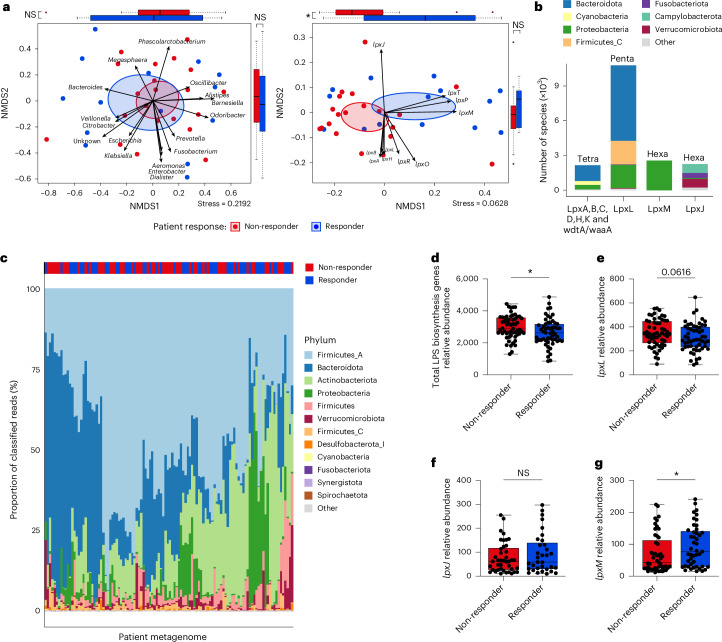
Patient responsiveness to anti-PD-1 therapy is associated with microbiome immunostimulatory hexa-acylated LPS. **a**, NMDS ordination of patient metagenomes with arrows indicating projected bacterial genera (left) and *lpx* encoding genes (right; NMDS axis 1 (NMDS1), *P = 0.01447) from patients with melanoma before anti-PD-1 therapy. *n* = 35. NMDS stress scores were calculated after 1,000 iterations or the best value achieved. Ellipses were drawn with a 95% confidence interval. Boxplots show the distribution of data points (coordinates) within NMDS1 and NMDS axis 2 (NMDS2). **b**, Taxonomic breakdown of *lpx* encoding bacteria stacked according to the encoded enzymes for the lipid A tetra-acylated backbone (left) and each level of lipid A acylation as indicated above each stacked bar. **c**, Phylum-level taxonomic composition of pre-treatment faecal metagenomes from 112 patients with melanoma. The relative abundance of each phylum is given as a proportion of total classified reads, and each phylum is indicated by the fill colour of the stacked bars. **d**–**g**, Relative abundance as CPM of total LPS biosynthesis genes (*P = 0.0218) (**d**) and of *lpxL* (penta-acylation) (**e**), *lpxJ* (hexa-acylation) (**f**) and *lpxM* (hexa-acylation) (**g**) genes (*P = 0.0453) within faecal metagenomes of responder (*n* = 55) and non-responder (*n* = 57) patients with metastatic melanoma before treatment with anti-PD-1. Two-tailed *t*-test with data presented as boxes extending from the 25th to 75th percentiles, bars at medians and whiskers from minimum to maximum in **a**. Two-tailed Mann–Whitney test with data presented as boxes from the 25th to 75th percentiles, bars at median and whiskers from minimum to maximum in **d**–**g**. NS, not significant. Source data

To explore the functional role of microbiota-derived LPS in responses to anti-PD-1 immunotherapy, we first analysed baseline gut microbiota composition before treatment using metagenomic sequences from a large-cohort study of anti-PD-1-treated patients with melanoma^1^. Non-metric multidimensional scaling (NMDS) ordination based on taxonomy and abundance of Gram-negative bacterial genera did not significantly segregate responders from non-responders (Fig. 1a, left). However, NMDS ordination based on function, specifically abundance of genes involved in LPS biosynthesis, significantly segregated responders from non-responders and revealed differential abundance of *lpxM*, which mediates lipid A hexa-acylation (Fig. 1a, right). Among genes involved in bacterial LPS assembly, *lpxA*, *B*, *C*, *D*, *H*, *K* and *wdtA/waaA* encode genes required for basic LPS assembly and tetra-acylation of lipid A, whereas *lpxL* facilitates LPS penta-acylation, and *lpxJ* and *lpxM* hexa-acylate LPS with differing acyl chain lengths and positions^14,15^.

**Extended Data Fig. 1:**
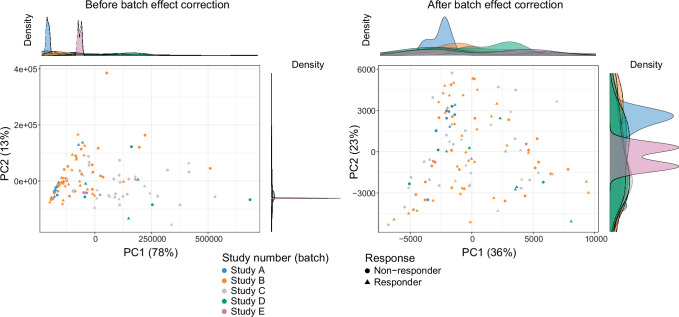
Batch corrections reduce variance from study and geographic location to reveal microbiome associations with anti-PD-1 response. Principal component analysis of the functional profile (KEGG OGs) of the pooled melanoma cohort before and after batch correction. Individual studies are shown in different colors. Density plots with different colors on the axes represent the sample distribution per study. Before batch correction samples were mostly clustered by study (left) while batch correction removed that effect (right). Permutational multivariate analysis of variance (PERMANOVA) results from the functional and taxonomic annotation before and after batch correction are shown in Supplementary Table 2.

**Extended Data Fig. 2:**
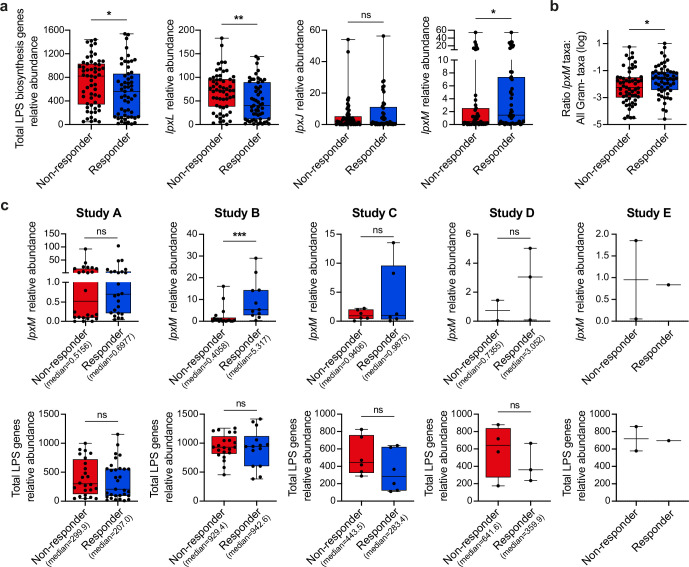
Relative abundances without batch correction of LPS-encoding genes in melanoma patient metagenomes prior to anti-PD-1 treatment. **a**, Relative abundance as gene count per million (GCPM) of total LPS biosynthesis genes (*P = 0.0413), *lpxL* (**P = 0.0072), *lpxJ*, and *lpxM* (*P = 0.0343), respectively (from left to right) in responder (n = 55) and non-responder (n = 57) metastatic melanoma patients. **b**, Ratio of *lpxM* encoding taxa to all other gram-negative taxa (*P = 0.0338) in indicated patient metagenomes. **c**, Relative abundance as GCPM of *lpxM* (upper row, Study B ***P = 0.0009) and total LPS biosynthesis genes (lower row) within metagenomes of responder and non-responder metastatic melanoma patients in individual studies. Two-tailed Mann-Whitney test with data presented as box from the 25th to 75th percentile, bar at median and whiskers from minimum to maximum (**a-c**). *P < 0.05, **P < 0.01, ***P < *0.001*. Source data

We next expanded our analyses by performing taxonomic and functional annotation on metagenomes from five published datasets encompassing 112 patients with melanoma treated with anti-PD-1 from seven clinical studies across four different countries^1,3,4,11,16^, batch correcting for variance introduced by study and geographic differences (Extended Data Fig. 1 and Supplementary Tables 1 and 2). It is worth noting that only samples taken before the start of immunotherapy from patients not taking probiotics, antibiotics or medications such as proton pump inhibitors and H2 receptor antagonists were included, as these treatments are known to have significant effects on the microbiome, complicating assessment of the patient’s baseline microbiota^3^. On a taxonomic level, a majority of the commensal Gram-negative species in patient gut microbiotas encoded *lpxL*-mediated capacity for penta-acylated LPS biosynthesis, dominated by members of the Bacteroidota phylum (Fig. 1b), which has previously been demonstrated to be immunoinhibitory^7,8^. While we observed considerable inter-patient variation on a taxonomic level (Fig. 1c), the abundance of total LPS biosynthesis genes was significantly higher in non-responders to anti-PD-1 therapy (Fig. 1d), as previously noted^4^. Among the genes involved in modification of lipid A, *pagL* (lipid A 3-O-deacylation), *pagP* (palmitoylation), *lpxE* and *lpxF* (dephosphorylation), *lpxL* (penta-acylation) and *lpxJ* and *lpxM* (different forms of hexa-acylation), only *lpxM* was significantly different between responders and non-responders (Fig. 1e–g), predominantly represented by Proteobacteria (homonym Pseudomonadota) (Fig. 1b). It is worth noting that these functional comparisons of microbiome LPS biosynthesis genes were consistent with those from pooled and per-study data before batch correction (Extended Data Fig. 2). These analyses confirmed a negative correlation between total gut microbiome LPS and immunotherapy response but uncovered a significant positive association between *lpxM*-mediated LPS hexa-acylation and favourable anti-PD-1 therapy outcomes.

### Functional metagenomics reveal hexa-LPS signature of response

**Fig. 2:**
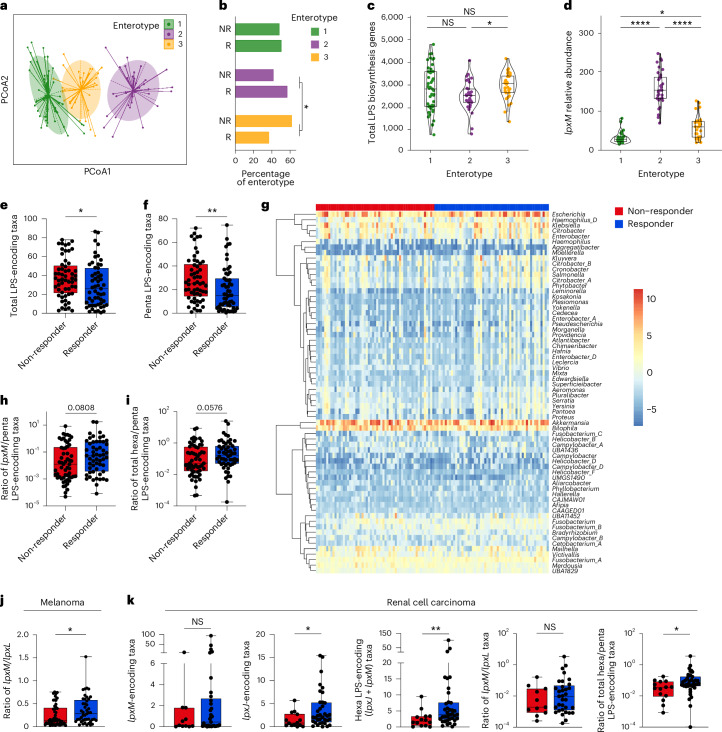
Functional metagenomic analyses indicate elevated hexa-acylated LPS ratios are favourable for patient responses to anti-PD-1 therapy. **a**, Functional enterotypes of gut microbiomes of patients with metastatic melanoma illustrated using JSD with PAM clustering and principal coordinate analysis (PCoA). **b**, Percentage of non-responding (NR) and responding (R) patients within each enterotype (*P = 0.03424). **c**,**d**, Relative abundances of total *lpx* encoding genes as CPM (2 versus 3, *P = 0.03) (**c**) and *lpxM* encoding genes within each patient enterotype (1 versus 2, ****P = 4.11 × 10^−15^; 1 versus 3, *P = 0.02; 2 versus 3, ****P = 1.70 × 10^−7^) (**d**). **e**,**f**, Total LPS-encoding taxa (*P = 0.0419) (**e**) and penta-acylated LPS-encoding taxa (**P = 0.0086) (**f**) as a percentage of classified reads. **g**, Centre-log ratio normalized relative abundance of *lpxJ-* and *lpxM*-encoding genera in patient faecal metagenomes clustered by rows (genera). **h**,**i**, Taxa predicted to produce *lpxM*-mediated (**h**) or *lpxJ-* and *lpxM*-mediated (**i**) total hexa-acylated LPS as ratios to *lpxL*-encoding penta-acylated LPS taxa. **j**, Ratio of *lpxM* to *lpxL* genes (*P = 0.0292) based on CPM within faecal metagenomes of patients with metastatic melanoma. **k**, Relative abundance as GCPM of (from left to right) *lpxM*, *lpxJ* (*P = 0.0356), total hexa-acylation (*lpxM* and *lpxJ*) (**P = 0.0086) encoding genes and indicated ratios of *lpxM* and total hexa-acylation encoding taxa (*P = 0.0240) to penta-acylation encoding taxa within faecal metagenomes of patients with RCC before treatment with anti-PD-1. The legend at the top of **g** also applies to **j** and **k**. Patients with metastatic melanoma, *n* = 112 in **a**–**j**. Patients with RCC, *n* = 51 in **k**. One-tailed two-proportions *Z*-tests without continuity correction in **b**. Kruskal–Wallis with post hoc Dunn test and Bonferroni false discovery rate correction with data presented as boxes from the 25th to 75th percentiles, bars at median and whiskers from minimum to maximum in **c** and **d**. Two-tailed Mann–Whitney test with data presented as boxes from the 25th to 75th percentiles, bars at median and whiskers from minimum to maximum in **e**, **f** and **h**–**k**. Source data

**Extended Data Fig. 3:**
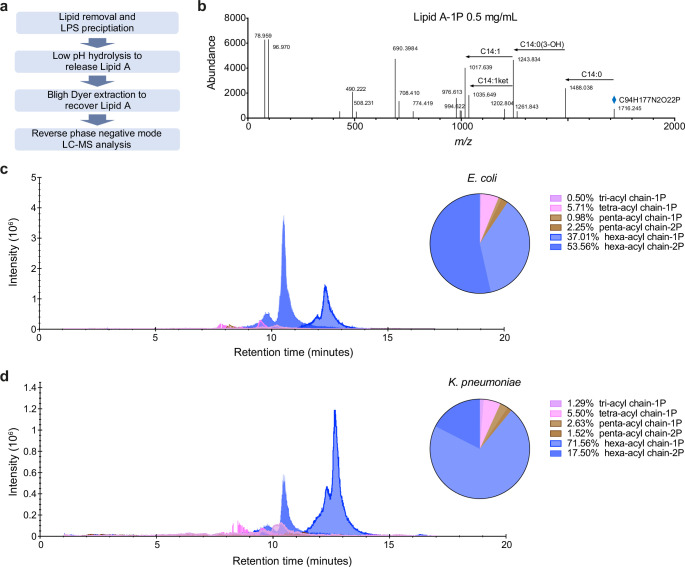
Genomically encoded lpxM accurately predicts lipid A hexa-acylation capacity of gut microbes. Two commensal isolates from our culture collection that encode *lpxM* were grown anaerobically, and **a**, lipid A was isolated from resulting cell pellets and analyzed using a customized LC-MS lipidomics method for characterization of lipid A acylation. **b**, MS/MS spectra of one characteristic hexa-acyl-1P ion (*m/z* 1716) of a purchased standard lipid A-1P from *Escherichia coli*. The characteristic acyl chain losses are annotated in the spectra as described by Sándor et al., 2016. **c**, Overlaid extracted ion chromatogram (EIC) of lipid A from mouse *E. coli* isolate LPS (left) and relative composition in terms of acylation and phosphorylation (right). **d**, Overlaid EIC of lipid A from mouse *Klebsiella pneumoniae* isolate LPS (left) and relative composition in terms of acylation and phosphorylation (right). Source data

To validate our findings using additional unbiased clustering methods, we identified three functional enterotypes (clusters of bacterial communities in the gut) using partitioning around medoids (PAM) clustering with a Jensen–Shannon divergence (JSD) distance metric calculated from the functional annotation profiles of the metagenomes of the 112 patients with melanoma (Fig. 2a). A majority of patients with functional enterotype 2 were responders to anti-PD-1 therapy, while most patients with functional enterotype 3 were non-responders (Fig. 2b). Consistent with our other analyses, while functional enterotype 3 was characterized by a significantly higher abundance of total LPS biosynthesis genes (Fig. 2c), functional enterotype 2 was characterized by a significantly higher abundance of *lpxM* (Fig. 2d). Although direct measurement of lipid A acylation in complex mixtures such as faeces remains challenging, as a proof of principle, we used isolates from our culture collection^17^ and confirmed by liquid chromatography–mass spectrometry that gut commensal Gammaproteobacteria that encode *lpxM* can produce hexa-acylated lipid A (Extended Data Fig. 3).

Using taxonomic classification paired with pangenome annotation of LPS biosynthesis genes, we also quantified the abundance of microbial taxa that encode *lpxL* penta-acylated versus *lpxJ* and *lpxM* hexa-acylated LPS. On a taxonomic level, both total LPS-encoding and penta-acylated LPS-encoding taxa were significantly higher in non-responders to anti-PD-1 (Fig. 2e,f). However, differential abundance of hexa-acylation was not dominantly mediated by a single genus or species of bacteria but rather a functional convergence on total LPS hexa-acylation capacity via multiple *lpxJ-* and *lpxM*-encoding taxa in the gut microbiota of responding patients (Fig. 2g). This resulted in ratios of hexa- to penta-acylated LPS-producing taxa that were more than doubled in responders (mean *lpxM*-to-penta ratios are Responder = 0.9238, Non-responder = 0.4217, and mean total hexa-to-penta ratios are Responder = 1.255, Non-responder = 0.6244) (Fig. 2h,i) with significantly higher *lpxM*-to-*lpxL* ratios at a functional (gene or open reading frame (ORF)) level (Fig. 2j). It is worth noting that we also functionally annotated and examined abundance of LPS biosynthesis genes in patients with renal cell carcinoma (RCC) from a single previous study^2^. Although *lpxM* also trended higher in this smaller cohort, we found in patients with RCC that *lpxJ*, which also mediates LPS hexa-acylation, was significantly more abundant in responders (Fig. 2k), in line with the increased abundance of *Akkermansia muciniphila* previously noted in this patient cohort^2^. This also resulted in an increased ratio of total hexa-to-penta-acylated LPS genes in patients responding to anti-PD-1 (Fig. 2k), raising the possibility that similar microbiome LPS signatures are associated with anti-PD-1 efficacy in other cancer types. Collectively, these data suggested that having an increased proportion of gut bacteria that encode hexa-acylated LPS is favourable for response to anti-PD-1 immunotherapy.

### Microbiome LPS and TLR4 are required for anti-PD-1 efficacy

**Fig. 3:**
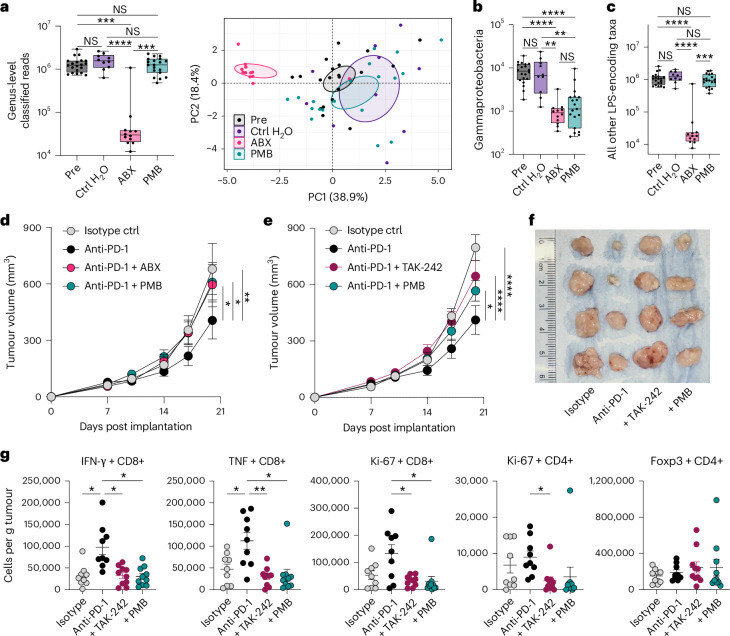
Microbiota-derived hexa-acylated LPS and host TLR4 are required for anti-PD-1-mediated anti-tumour immunity. Mice were treated with PMB, broad-spectrum antibiotics (ABX) or control drinking water (control (Ctrl) H_2_O) for 2 weeks. **a**, Abundance of bacterial taxa measured in mouse faecal metagenomes as the number of classified reads at genus level normalized to faecal weight (left) (Pre versus ABX, ***P = 0.0001; Ctrl H_2_O versus ABX, ****P < 0.0001; ABX versus PMB, ***P = 0.0002) and principal component analysis of the treatment groups at the phylum level showing the differential effects on the mouse gut microbiota (right). Ellipses were drawn with a 99% confidence interval. **b**,**c**, Relative abundance of Gammaproteobacteria (Pre versus ABX, ****P < 0.0001; Pre versus PMB, ****P < 0.0001; Ctrl H_2_O versus ABX, **P = 0.0010; Ctrl H_2_O versus PMB, **P = 0.0029) (**b**) and LPS-encoding non-Gammaproteobacteria (Pre versus ABX, ****P < 0.0001; Ctrl H_2_O versus ABX, ****P < 0.0001; ABX versus PMB, ***P = 0.0003) (**c**) in the faecal metagenomes of treated mice shown as the mean of classified reads normalized to faecal weight. Pre, *n* = 20; Ctrl H_2_O, *n* = 10; ABX, *n* = 12; and PMB, *n* = 19 mice per group pooled from 2 experimental repeats in **a**–**c**. **d**, Indicated mice were pre-treated for 2 weeks with ABX or PMB in their drinking water. MC38 colorectal adenocarcinoma cells were subcutaneously implanted, and tumour growth was measured at serial time points following tumour implantation. Indicated animals were treated with anti-PD-1 i.p. starting at day 10 after tumour implantation (anti-PD-1 versus Isotype ctrl, **P = 0.0054; anti-PD-1 versus Abx mix + anti-PD-1, *P = 0.0478; anti-PD-1 versus PMB + anti-PD-1, *P = 0.0318). Data shown were pooled from 2 experimental repeats with *n* = 10 mice per group. **e**, MC38 colorectal adenocarcinoma cells were subcutaneously implanted into PMB pre-treated or untreated mice. Measurements were taken at indicated time points post implantation. Anti-PD-1 was administered i.p. starting at day 10 after tumour implantation, and the TLR4 inhibitor TAK-242 was administered i.p. during anti-PD-1 therapy (anti-PD-1 versus Isotype ctrl, ****P < 0.0001; anti-PD-1 versus PMB + anti-PD-1, *P = 0.0162; anti-PD-1 versus anti-PD-1 + TAK242, ****P < 0.0001). Data shown were pooled from 2 experimental repeats with *n* = 10 mice per group. **f**, Representative tumour pictures. **g**, Representative flow cytometry measurements of indicated T-cell phenotypes from tumours in **f** (exact *P* values provided in Source Data Fig. 3). Kruskal–Wallis test comparing all to all with data presented as box from the 25th to 75th percentiles, bars at median and whiskers from minimum to maximum in **a**–**c**. Two-way ANOVA comparing all to anti-PD-1 in **d** and **e**, and Brown–Forsythe with post hoc Dunnett’s test comparing all to anti-PD-1 in **g**, with bars and error representing mean and s.e.m. Source data

**Extended Data Fig. 4:**
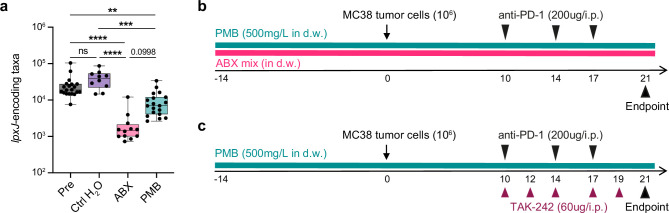
Selective depletion of hexa-acylated LPS and TLR4 signalling by polymyxin B and TAK-242. Mice were treated with polymyxin B (PMB), broad-spectrum antibiotics (ABX) or control drinking water for two weeks. **a**, Abundance of *lpxJ*-encoding bacterial taxa (Pre vs ABX, ****P < 0.0001; Pre vs PMB, **P = 0.0087; Ctrl H2O vs ABX, ****P < 0.0001; Ctrl H2O vs PMB, ***P = 0.0009) measured in mouse fecal metagenomes, shown as average number of classified reads at species level normalized to fecal weight, before and after treatment. n = 20 (Pre), 10 (Ctrl H_2_O), 12 (ABX) and 19 (PMB) mice per group pooled from 2 experimental repeats. **b** and **c**, Schematics for *in vivo* ABX, PMB and TAK-242 (TLR4 inhibitor) treatment regimens in the context of anti-PD-1 immunotherapy. Kruskal-Wallis test (**a**). **P < 0.01, ***P < 0.001, ****P < *0.0001*. Source data

To investigate potential mechanisms by which the acylation of microbiota-derived LPS might impact anti-PD-1 responses, we explored targeted oral antibiotic treatments to alter populations of Gram-negative gut bacteria. We treated mice with control drinking water, a cocktail of broad-spectrum antibiotics, or polymyxin B (PMB), a narrow-spectrum antibiotic known to both inhibit LPS signalling and deplete Gram-negative bacteria by binding the lipid A region of LPS^18^. We then performed shotgun metagenomic sequencing of faecal samples before and after treatment to assess effects on the relative abundance of different taxa in the gut microbiota of treated mice. Overall, PMB treatment significantly preserved microbial abundance and microbiota composition compared to treatment with broad-spectrum antibiotics (Fig. 3a). However, while both antibiotic regimens were equally effective at depleting Gammaproteobacteria (Fig. 3b), PMB did not significantly deplete other LPS-encoding Gram-negative taxa, with the exception of *lpxJ*-encoding taxa, compared to control drinking water (Fig. 3c and Extended Data Fig. 4a). By contrast, broad-spectrum antibiotics significantly depleted all LPS-encoding bacteria (Fig. 3c and Extended Data Fig. 4a), in line with their total reduction of bacterial reads (Fig. 3a). Using this selective depletion of hexa-acylated LPS-encoding Gammaproteobacteria by PMB, we investigated how altering LPS acylation ratios in the gut microbiome affects anti-PD-1 efficacy in a mouse model of tumour immunotherapy. As a proof of principle, we used the well-established anti-PD-1-responsive, syngeneic MC38 colorectal adenocarcinoma tumour model^19^ and pre-treated mice with PMB or a broad-spectrum antibiotic cocktail in their drinking water starting 2 weeks before subcutaneous tumour implantation (Extended Data Fig. 4b). As previously reported^1,2^, broad-spectrum antibiotics significantly abrogated anti-PD-1-induced tumour reduction; however, targeted microbiome depletion with PMB equally abolished anti-PD-1 efficacy (Fig. 3d). These observations together revealed that selective reduction of gut bacteria capable of producing immunostimulatory hexa-acylated LPS was sufficient to disrupt anti-PD-1-mediated anti-tumour responses.

**Extended Data Fig. 5:**
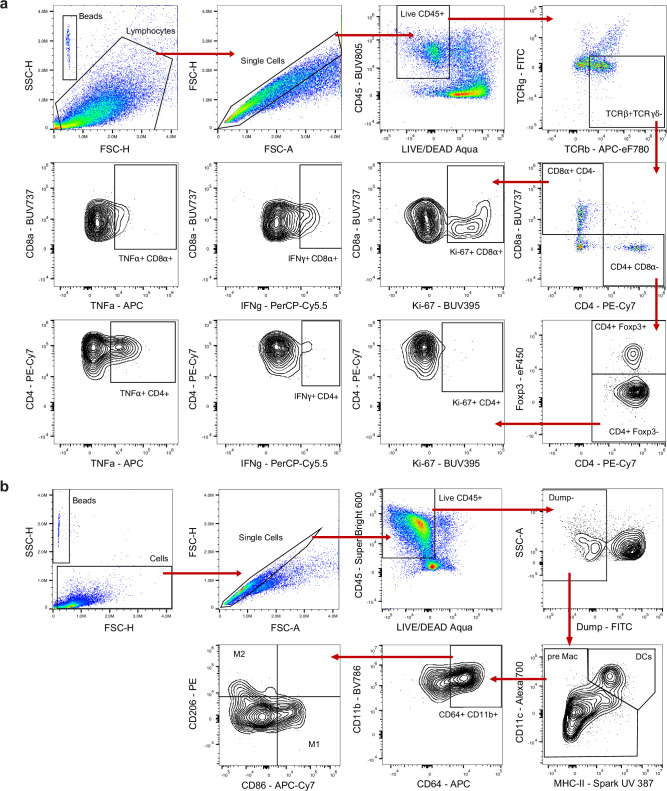
Flow cytometry phenotyping of immune cells from treated tumor-bearing mice. Representative T cell **a**, and myeloid cell **b**, gating strategies for data in Figs. 3 and 4 and Extended Data Figs. 6 and 8.

**Extended Data Fig. 6:**
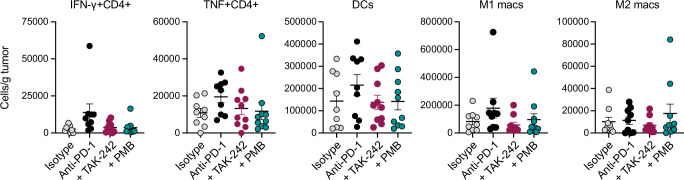
Effects of TLR4 inhibitor TAK-242 on tumor-infiltrating immune cells. MC38 colorectal adenocarcinoma cells were subcutaneously implanted into PMB pre-treated or untreated mice. Measurements were taken at indicated time points post implantation. Anti-PD-1 was administered i.p. starting at day 10 after tumor implantation, and the TLR4 inhibitor TAK-242 was administered i.p. during anti-PD-1 therapy. Flow cytometry measurements of indicated immune cells isolated from tumors of treated mice are shown. Data shown were pooled from 2 experimental repeats with n = 10 mice per group (exact p-values provided in Source Data Extended Data Figures). No differences were statistically significant by Kruskal-Wallis test. Source data

As TLR4 is the canonical host receptor for LPS and is required for a majority of its endotoxic and immunomodulatory effects^20^, we next investigated the contribution of host TLR4 signalling to anti-PD-1 responses. We treated tumour-bearing wild-type mice with the small-molecule TLR4 inhibitor TAK-242 only during anti-PD-1 therapy and examined the effect of treatment on tumour growth (Extended Data Fig. 4c). It is worth noting that temporally controlled inhibition of TLR4 during anti-PD-1 treatment alone resulted in significant loss of treatment efficacy (Fig. 3e,f). This was accompanied by reduced infiltration of IFNγ^+^ and TNF^+^ CD8^+^ T cells and proliferating Ki67^+^ effector T cells within tumours of TAK-242-treated animals (Fig. 3g and Extended Data Fig. 5) without significant effects on other immune cell populations (Extended Data Fig. 6). Taken together, these data indicated that stimulation of host TLR4 by microbiota-derived hexa-acylated LPS was required for effective anti-tumour immunity in response to anti-PD-1.

### Hexa-LPS stimulates host immunity and enhances immunotherapy

**Fig. 4:**
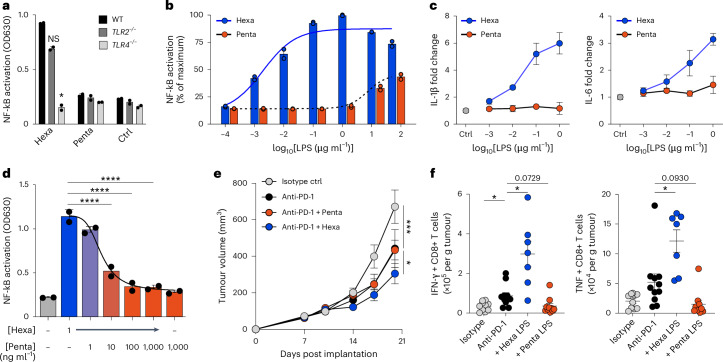
Oral administration of hexa-acylated LPS enhances the efficacy of anti-PD-1 therapy. **a**–**d**, Wild-type, *TLR2*^*−/−*^ or *TLR4*^*−/−*^ NF-kB reporter THP-1 (human monocyte/macrophage) cells were incubated with the indicated doses of hexa- or penta-acylated LPS for 24 h. Data are from two biological replicates per concentration of LPS and are representative of at least three experimental replicates. **a**, NF-kB activation in response to 1 ng ml^−1^ hexa-acylated LPS or 1 µg ml^−1^ of penta-acylated LPS (Hexa LPS WT versus *TLR4*^*−/−*^, *P = 0.0274). **b**, NF-kB activation dose–response with the indicated doses of hexa- or penta-acylated LPS. **c**, IL-1β (left) and IL-6 (right) secretion by THP-1 cells after 24 h treatment with the indicated doses of LPS, shown as fold increase over media controls. **d**, NF-kB activation with the indicated combined doses of hexa- and penta-acylated LPS (1 ng Hexa versus +10, +100 and +1,000 ng Penta, ****P < 0.0001). **e**, MC38 colorectal adenocarcinoma cells were subcutaneously implanted into wild-type mice, and measurements were taken at indicated time points post implantation. Indicated animals were treated with anti-PD-1 i.p. and/or oral hexa- or penta-acylated LPS or control drinking water starting at day 10 after tumour implantation (anti-PD-1 versus Isotype ctrl, ***P = 0.0002; anti-PD-1 versus anti-PD-1 + Hexa, *P = 0.0318). Data shown were pooled from 2 experimental repeats with *n* = 10 mice per group. **f**, Flow cytometry measurements of IFNγ (anti-PD-1 versus Isotype ctrl, *P = 0.0325; anti-PD-1 versus anti-PD-1 + Hexa, *P = 0.0423) and TNF (anti-PD-1 versus anti-PD-1 + Hexa, *P = 0.0352) producing CD8^+^ T cells infiltrating tumours of mice in **e** with *n* = 5 mice per group. Brown–Forsythe and Welch ANOVA comparing all to WT in **a**. One-way ANOVA with a Dunnett’s multiple comparisons test in **d**. Two-way ANOVA comparing all to anti-PD-1 in **e** and Brown–Forsythe with post hoc Dunnett’s test comparing all to anti-PD-1 in **f**, with bars and error representing mean and s.e.m. Source data

**Extended Data Fig. 7:**
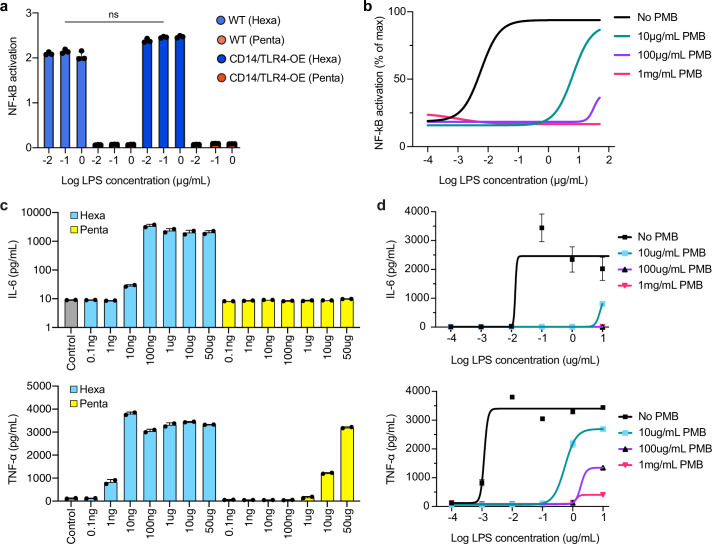
Contributions of host receptors and LPS acylation states to immune activation. **a**, NF-kB reporter human THP-1 cells that do not (WT) or do over-express CD14 and TLR4 (CD14/TLR4-OE) were incubated with the indicated doses of hexa- or penta-acylated LPS for 24 h. Representative data from n = 3 biological replicates are shown from one of two experimental repeats. No significant differences between WT and CD14/TLR4-OE were calculated by two-tailed Mann-Whitney tests comparing equivalent doses and types of LPS. **b**, WT NF-kB reporter THP-1 cells were incubated with the indicated doses of hexa-acylated LPS and polymyxin B (PMB) for 24 h. NF-kB activation is expressed as a percentage of maximum. The means of n = 2 biological replicates are plotted in representative plots from one of three experimental repeats. Mouse RAW 264.7 macrophages were incubated with the indicated doses of hexa- or penta-acylated LPS without (**c**) or with (**d**) indicated concentrations of polymyxin B (PMB) for 24 h. Cytokine secretion was measured by cytometric bead arrays from culture supernatants. **c**, IL-6 (top) and TNF-α (bottom) secretion after 24 h of the indicated doses of hexa- or penta-acylated LPS. **d**, IL-6 (top) and TNF- α (bottom) secretion after 24 h of the indicated doses of hexa-acylated LPS and PMB. The means of n = 2 biological replicates are plotted in representative plots from one of three experimental repeats. For all plots, data are presented as mean ± SD. Source data

Given that the acylation state of LPS lipid A has been shown to directly impact immune signalling through TLR4^6,21^, we quantified the relative potency of hexa-acylated and penta-acylated LPS at stimulating NF-kB activation via TLR4 in both human monocyte/macrophage and mouse macrophage cell lines. As expected, in human monocytes we found that TLR4, but not TLR2, was required for LPS-mediated NF-kB activation, and stimulation with hexa-acylated LPS resulted in substantially more activation than penta-acylated LPS (Fig. 4a). By contrast, expression of CD14, a receptor that also binds LPS but does not discriminate lipid A acylation states, did not affect differential responses to hexa- and penta-acylated LPS (Extended Data Fig. 7a). Hexa-acylated LPS purified from a Gammaproteobacterium was 10,000 times more potent at stimulating NF-kB activation than penta-acylated LPS from a Bacteroidota species (Fig. 4b), resulting in higher cytokine secretion (Fig. 4c). This was blocked by PMB in a dose-dependent manner (Extended Data Fig. 7b), and although the LPS response between human and mouse TLR4/MD2 has been shown to differ^8^, similar effects on cytokine secretion (as a proxy of NF-kB activation) were also observed in a mouse macrophage cell line (Extended Data Fig. 7c,d). It is worth noting that in human monocyte/macrophages, altering the ratio of hexa- and penta-acylated LPS via the addition of low, non-stimulatory concentrations of penta-acylated LPS significantly decreased the ability of hexa-acylated LPS to activate NF-kB (Fig. 4d), confirming that ratios of stimulatory to inhibitory LPS can dictate immune activation.

**Extended Data Fig. 8:**
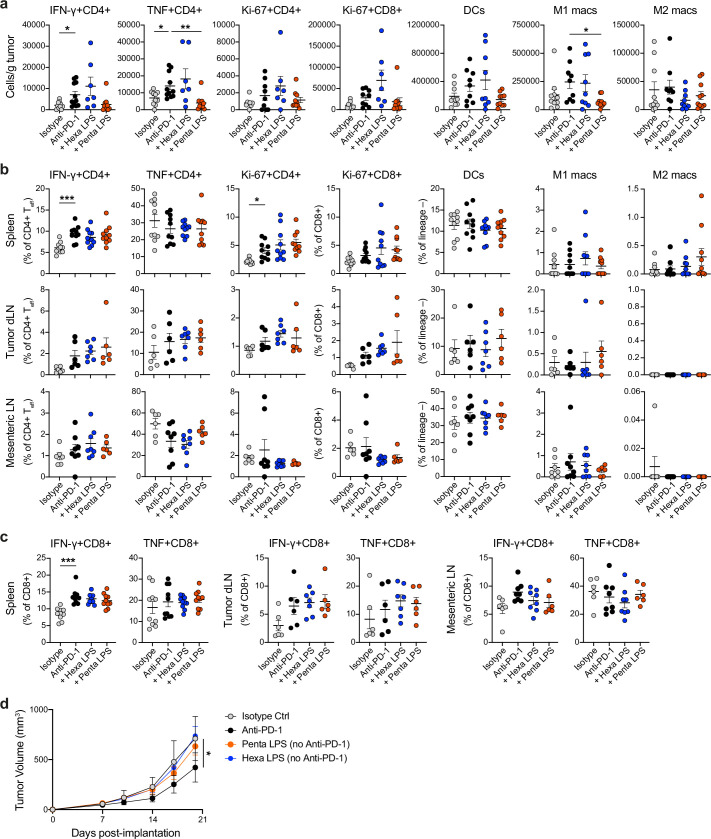
Effects of hexa- and penta-acylated LPS on tumor-infiltrating and peripheral immune cells. MC38 cells were subcutaneously implanted into C57BL/6 mice. Indicated animals were treated with anti-PD-1 i.p. with or without oral hexa- or penta-acylated LPS in drinking water starting at day 10 after implantation. **a-c**, Flow cytometry measurements of immune cells isolated from indicated tissues of treated mice at day 21 after tumor implantation (exact p-values provided in Source Data Extended Data Figures). Data shown were pooled from 2 experimental repeats with n = 6-10 mice per group, depending on tissue, with each data point representing one mouse. Kruskal-Wallis test comparing all to anti-PD-1. **d**, Tumor measurements from mice treated with anti-PD-1 i.p. or oral hexa- or penta-acylated LPS starting at day 10 after implantation (anti-PD-1 vs Isotype Ctrl, *P = 0.0271). Data shown are from 1 experiment with n = 10 mice per group. Two-way ANOVA comparing all to isotype control. For all plots, data are presented as mean ± SEM. *P < 0.05, **P < 0.01, ***P < *0.001*. Source data

To examine the effects of increasing the ratio of immunostimulatory hexa-acylated LPS in the gut, tumour-bearing mice were supplemented with a low dose of hexa- or penta-acylated LPS in their drinking water during anti-PD-1 treatment. Boosting levels of hexa-acylated LPS significantly improved the efficacy of anti-PD-1 therapy, resulting in decreased tumour burden (Fig. 4e), while oral supplementation with penta-acylated LPS did not significantly affect anti-PD-1 efficacy. In addition, when examining the tumour-infiltrating immune cells from these mice, we observed a significant increase in effector cytokine-secreting CD8^+^ T cells infiltrating the tumours of hexa-acylated LPS-supplemented mice (Fig. 4f), with a trend towards decreased immune activation in penta-acylated LPS-treated mice (Fig. 4f and Extended Data Fig. 8a). Oral LPS did not result in any signs of inflammation or systemic immune activation, as no significant differences were observed in spleen or peripheral lymph nodes in mice treated with oral LPS and anti-PD-1 compared to anti-PD-1 alone (Extended Data Fig. 8b,c). This suggests that hexa-acylated LPS augmentation of anti-PD-1-mediated immunity is restricted to the tumour. Neither hexa- nor penta-acylated LPS affected tumour growth in the absence of anti-PD-1 (Extended Data Fig. 8d). Collectively, these findings show that an increased relative abundance of hexa-acylated LPS in the gut can support the immune activation required for robust anti-PD-1 efficacy in both patients and mouse models.

## Discussion

Improving response rates to ICI is essential for improving survival of patients. In this initial study, we functionally annotated a large cohort of patient microbiota profiles to uncover the role of gut bacteria in ICI efficacy. Although differences in patient population based on geographic location significantly contribute to variance at a taxonomic level (even after batch correction), this source of variation contributed far less at a functional level (Extended Data Fig. 1 and Supplementary Table 2), supporting the use of our functional metagenomics approach to see past taxonomic heterogeneity to an underlying mechanism involving LPS acylation. By exploring this mechanism in animal models, we were able to demonstrate that hexa-acylated LPS increases the efficacy of anti-PD-1 ICI treatment in a TLR4-dependent manner, cautioning against use of PMB and related antibiotics in patients undergoing immunotherapy treatments and highlighting actionable potential avenues to therapeutically predict and pharmacologically improve patient outcomes.

It is important to note that inferred LPS biosynthesis capacity based on taxonomic identification and pangenome annotations did not always match our taxonomy-independent functional analyses at the level of LPS biosynthesis genes. This highlights potential limitations of taxonomy-based analyses for inferring function and may explain how meta-analyses in previous studies did not identify differential contributions of LPS hexa- and penta-acylation to immunotherapy responses. In addition, current databases and methods of functional annotation of microbiome genes are far from complete and comprehensive, necessitating our use of multiple approaches for both taxonomic and functional analyses to derive biological meaning. Methods to accurately measure different lipid A acylation states in complex environments such as patient stool samples also merit further development and optimization to determine the degree to which metagenomic profiles can reflect the heterogenous mixture of lipid A variants in the gut.

The ability of human TLR4/MD2 to discriminate LPS of distinct acylation states differs from that of mouse TLR4/MD2^8^, and it has been previously shown that hexa-acylated LPS potently activates human TLR4/MD2, whereas hypo-acylated tetra- and penta-acylated LPS poorly activate or antagonize human TLR4/MD2 activation driven by hexa-acylated LPS^22,23^. Thus, while the mouse model was critical to demonstrate a requirement for hexa-acylated LPS/TLR4 signalling in anti-tumour PD-1 responses, and the ability of orally administered hexa-acylated LPS to augment such responses, we complemented our metagenomic analyses of human anti-PD-1 responses with human macrophage experiments to explore the relative activating versus immunosilencing properties of hexa- and penta-acylated LPS in vitro. Further optimization of ‘humanized’ mouse models holds promise for better in vivo characterization of the possible mechanisms of hexa-acylated LPS-mediated immunotherapy enhancement. For example, the NSG TLR4^−/−^ mouse model reconstituted with human haematopoietic stem cells^24^ can be used to test effects of hexa-acylated LPS on immune cells expressing human TLR4, although this system will not account for possible roles of TLR4 activation in intestinal epithelial cells and other stromal cell types. Mice expressing only human TLR4/MD-2 have also been generated, but only male mice can be used due to integration on the Y chromosome^25^.

Other microbiota-derived metabolites, including *Bifidobacterium* inosine^26^, *Enterococcus* muropeptides^27^ and bacterial flagellin^28^, have also been implicated in modulating ICI treatment response. While it is currently unknown to what degree gut LPS signalling interfaces with signals from other microbiome-derived immunostimulatory metabolites, simultaneous activation of different pattern recognition receptors has been shown to result in synergistic immunostimulation^29,30^. It is therefore likely that multiple microbiota-mediated mechanisms that modulate treatment response coexist and interact to define patient outcomes, perhaps contributing to the divergent taxonomic associations described by previous studies. In addition, host factors are likely to play a role in this mechanism of immunotherapy modulation. For instance, differential expression of host intestinal alkaline phosphatase has been shown to affect the strength of the TLR4 signal^31^, and this represents just one of many currently uncharacterized patient factors that could be explored in the future.

Numerous other aspects of the microbiome LPS–immunotherapy interaction also warrant further studies, including the contribution of TLR4 in different cell types to anti-PD-1 efficacy. Additional tumour models, such as the YUMM2.1 melanoma model and the CT26 microsatellite-stable colorectal carcinoma model, may shed light on the broad applicability of hexa-acylated LPS for enhancing anti-PD-1 efficacy in other cancer types. In addition, as shown by the heterogeneity of Proteobacteria abundance seen in human patients in Fig. 1c, the levels of LPS tolerated in the gut can be quite high. This is in stark contrast to the relatively low concentrations (1–4 ng per kg body weight) of LPS needed in the circulation to cause pathology. Several studies in rodents have indeed demonstrated that oral LPS administration at doses up to 1 mg kg^−1^ does not cause any inflammation, pathology or associated toxicity and may instead have neuroprotective effects^32^. A better understanding of how and where gut-derived microbial metabolites engage host signalling pathways to determine clinical response to ICI immunotherapies will enable design of new therapeutic approaches to overcome microbiota-driven heterogeneity in immunotherapy outcomes and allow better prognostication to benefit patients with cancer.

## Methods

We confirm that our research complies with all relevant ethical regulations.

### Curation of publicly available datasets

We retrieved human metagenomic data from six publicly available datasets^1–4,11,16^ through the National Center for Biotechnology Information (NCBI) Sequence Read Archive (SRA) using the accession numbers ERP127050, SRP339782, SRP197281, SRP116709 and SRP115355 (ERP104577 for RCC) using NCBI sra-tools (v2.11.2). These publicly available cohorts are shown in Supplementary Table 1 (Supplementary Table 3 for RCC). We included only those samples in our study that were collected at baseline, that is, before starting the therapy. We excluded any samples from patients who received combination therapy (that is, anti-CTLA4 along with anti-PD1 or IFNγ with anti-PD1) and/or had a recent history of proton pump inhibitor, H_2_ receptor antagonist or antibiotic usage whenever metadata for these parameters was available. We classified patients into responder and non-responder groups according to the outcomes reported in the respective studies above. Patients classified as having stable disease, partial response or complete response were considered responders, and patients with progressive disease were considered non-responders for the purposes of our analyses.

We are aware that working with public datasets may introduce biases and artificial variability into the analyses due to nonuniformity of the data structure. It is noteworthy that in our study we did not directly use the processed count or abundance matrices, which are known to contribute to batch effects. Along with the baseline filtering criteria mentioned above, we also implemented a uniform stringent quality control and downstream analysis method for all the samples at individual raw sequencing read levels, as detailed below. Thus, all the pooled sequencing samples were treated as part of one experiment, and the source of the sample (that is, study and country) was used as a covariate, similar to the original studies.

### Quality control and preprocessing

The pre-processing pipeline consists of three main stages: (1) initial quality control by removing low-quality reads (quality score <Q30), reads <75 bp and Illumina adapters from the forward and reverse reads using Trimmomatic software (v0.39)^33^; (2) DNA contamination removal using Bowtie 2 (v2.3.5.1)^34^ in sensitive mode, removing host DNA from humans (GRCh38) and mice (GRCm39) and non-host phiX174 Illumina spike-in; and (3) checking the FastQC (http://www.bioinformatics.babraham.ac.uk/projects/fastqc/) results and removing any samples that had >50% read duplication from the human-associated metagenomes. FastQC results were aggregated with multiqc (v1.11) for easy manual checking.

### Contig assembly, gene-calling and functional annotation

Quality-trimmed host decontaminated paired-end metagenomic reads were assembled individually using metaSPAdes (v3.15.4)^35^ to build contigs. Single-end samples (RCC and cohorts; Supplementary Table 3) were assembled using MEGAHIT (v1.2.9)^36^. Contigs from individual samples were concatenated into a single file, and contigs smaller than 500 bp were discarded. To make a non-redundant contig database, 100% identical contigs were clustered, and a representative longer contig was kept using cd-hit-est from CD-HIT program (v4.8.1)^37,38^. Prodigal (v2.6.3)^39^ was used in ‘meta’ mode for the ORF and translated protein prediction from the non-redundant contig collection. The script sqm_annot.pl from SqueezeMeta software (v1.5.2)^40^ was used to assign KEGG (Kyoto Encyclopedia of Genes and Genomes) orthology and corresponding microbial taxa to the predicted protein sequences. The databases were downloaded, using the script make_databases.pl from SqueezeMeta. In brief, functions were assigned using Diamond (v2.0.15.153)^41^ blastp alignments of the proteins against the KEGG^42^ and NCBI non-redundant^43^ databases using the lowest common ancestor^44^ methods. This way each protein sequence from the metagenomes was assigned to a function (KEGG) and bacterial taxa, and thus a connection between function and taxonomy was established. To quantify the predicted genes in the samples across the metagenomes, we used the Salmon tool (v1.8.00)^45^. First, the predicted ORFs were indexed, and then reads (paired end or single end) from individual samples were mapped onto the indexed Salmon database.

### Analyses of patient metagenomes

Metagenomes from patients with melanoma or RCC were curated and quality controlled as described above. For gene-level relative abundance, undetected (zero value) samples were excluded, and all non-zero values were reported. In patients with RCC, the sample numbers for hexa-acylated LPS were as follows: *lpxM*, *n* = 44; *lpxJ*, *n* = 50; and total (*lpxM* + *lpxJ*), *n* = 51. A KEGG orthologous group present in less than five samples was considered as sporulating and/or misannotation and removed from the analysis. Relative abundance of the predicted LPS biosynthetic genes (ORFs) from the patient metagenomes was compared between ‘Responder’ and ‘Non-responder’ through a single comparison, and therefore there was no need for a multiple testing correction. Before functional analysis, we used ComBat-Seq (v3.48.0)^46^ to correct the batch effects from the functional profile of the cohort of patients with melanoma. ComBat-Seq was run on the read count matrix with study metadata as batch and treatment response as group (full model).

Taxonomic profiling was performed with Kraken v2.1.2^47^ and Bracken v2.6.2 (10.7717/peerj-cs.104) using a custom database built from representative genomes of the human gut microbiota that were re-annotated using the Genome Taxonomy Database v2.1. This database is available via Zenodo at 10.5281/zenodo.7319344 (ref. ^48^). After analysis with Kraken2/Bracken2, we used ConQuR (v1.2.0)^49^ to correct for batch effects between studies. ConQuR was run on the OTU read count matrix using a penalized fitting strategy (logistic_lasso=T, quantile_type=“lasso”, interplt=T) with study metadata as the batch variable and the response as a covariate. The resulting batch-corrected read count matrix was analysed in R (v4.3.1).

### Genomic prediction of bacterial LPS structure

To predict which bacterial species of the human gut microbiota produce LPS, *lpx* operon genes were identified from functional pangenomes of 3,006 bacterial species using the ‘feature_search’ module of the MGBC-Toolkit v1.0^17^. *lpx* genes were identified using both the InterPro and EggNOG functional schemata. Accessions for searched *lpx* genes are shown in Supplementary Table 4.

For each pangenome, genes that were encoded by at least 50% of the constituent genomes were considered to be encoded by that bacterial species. As most genes could be annotated using both InterPro and eggNOG, species that were identified as encoding a gene by at least one schema were included in analyses. All annotations were manually reviewed in context of the published literature and known structures for LPS. Following this review, only the InterPro database was used for *lpxM* annotation as eggNOG over-identified these genes in taxa which are known not to encode them.

LPS structure was then predicted using these annotation data according to the KEGG reference pathway for LPS biosynthesis. Species encoding *lpxA*, *C*, *D*, *B* and *K* were considered to produce lipid IVA and were thus defined as total Gram-negative LPS-encoding taxa (*n* = 884), while taxa that additionally encoded *kdtA/waaA* were considered to produce KDO2-lipid IVA (*n* = 827). Taxa that further encoded *lpxL* and either *lpxM* or *lpxJ* were considered to have the capacity to produce hexa-acylated LPS (*lpxLM*, *n* = 127; *lpxLJ*, *n* = 112), while taxa that further encoded *lpxL* but not *lpxM* or *lpxJ* were defined as penta-acylated LPS producers (*n* = 538). LPS-encoding taxa that did not encode *lpxL* were defined as tetra-acylated LPS producers (*n* = 107).

### Data normalization

Gene abundance of the single melanoma^1^, and RCC cohort was expressed in the form of gene count per million (GCPM).

The GCPM within the sample was calculated as follows:

$${{\rm{GCPM}}}_{i}=\frac{\left({q}_{i}/{l}_{i}\right)}{{\sum }_{j}\left({q}_{j}/{l}_{j}\right)}\times {10}^{6}$$

where *q*_*i*_ denotes the number of raw reads mapped to the predicted gene, *l*_*i*_ is the gene length, and ∑_*j*_(*q*_*j*_/*l*_*j*_) corresponds to the sum of mapped reads to the predicted gene normalized by gene length. Thus, GCPM accounts for gene length and sequencing depth and facilitates comparisons across samples. As the batch correction was performed on the raw read counts of the pooled cohort of patients with melanoma, we used a slightly different method to normalize and scale our data. The batch-corrected count matrix was log-transformed and added with a pseudo count of 10^−6^ followed by scaling to count per million (CPM) value.

The CPM within the sample was calculated as follows:

$${{\rm{CPM}}}_{i}=\frac{{A}_{i}}{{\sum }_{i=1}^{n}{A}_{i}}\times {10}^{6}$$

where *A*_*i*_ is the batch-corrected number of reads mapped to the predicted gene and *n* is the total number of predicted genes.

### Clustering and functional enterotype analysis

Functional enterotyping and between-class analyses were performed according to the procedure described by Arumugam et al.^50^ Data generated by functional annotation with the KEGG database (KEGG orthologs) were used to calculate the JSD among samples. The PAM clustering algorithm was applied to cluster the relative abundance of functional profiles of genus or orthologous groups. The number of optimal clusters was estimated using the Calinski–Harabasz index using the clusterSim package in R according to a previously described method^51^. The between-class analysis was performed to identify the major drivers of the metagenome functions and support clustering the orthologous groups abundance profiles into clusters. The between-class analysis is a type of ordination with instrumental variables with the highest effect sizes that maximize the separation between classes of this variable. In this study, the instrumental variable was cluster classification using PAM clustering, obtained from KEGG orthologous groups, which contributed the maximum to the principal coordinates, identified based on their eigenvalues. The analysis was performed using the ade4 package in R.

### Mice

In vivo tumour experiments were performed using 9-week-old female C57BL/6 mice purchased from Charles River. Experiments were conducted in the University of Cambridge UBS Anne McLaren facility in accordance with UK Home Office guidelines and were approved by the University of Cambridge Animal Welfare and Ethical Review Board (PPL number PP3638130). Mice were kept under a consistent 12 h light–dark cycle (7 a.m. to 7 p.m.), ambient temperature (20–24 °C) and humidity 45–65%, as per the UK Home Office guidelines. All mice were fed Scientific Diets SAFE 105 rodent diet ad libitum.

### Treatment of mice with antibiotics

Mice were treated with PMB (500 mg l^−1^) (APExBio, C3090-APE) or a broad-spectrum antibiotic solution (ABX: ampicillin (1 mg ml^−1^) (Cayman Chemical, 14417-25g-CAY), streptomycin (3 mg ml^−1^) (Sigma-Aldrich, S9137-25G), and colistin (1 mg ml^−1^) (BioServ UK Limited, BS-9867A-5G)) in sterile drinking water. Treatment was started 2 weeks before tumour implantation, and antibiotic water was changed twice a week.

### Shotgun sequencing of mouse metagenomes

Mouse faecal pellets were collected immediately after arrival from the supplier, termed ‘Pre’ samples. Additional faecal samples were collected after 2 weeks of treatment with broad-spectrum antibiotics, PMB or control drinking water. For broad-spectrum antibiotic-treated mice, treatment was discontinued for 1 day before sample collection to allow for some bacterial DNA to be recovered. A detailed description of the mouse metagenomic samples has been provided in Supplementary Table 5. Mouse faecal genomic DNA was extracted using the FastDNA SPIN kit for soil (MP Biomedicals, 116560200) per the manufacturer’s instructions and stored in a −80 °C freezer. Shotgun metagenomic sequencing was carried out at GENEWIZ from Azenta Life Sciences on an Illumina NovaSeq -X platform. Each sample was sequenced at a minimum depth of 20 million reads (minimum 10 million paired-end reads) with a 150-nucleotide read length. Sequences are deposited in NCBI SRA under Bioproject ID PRJNA1171992.

### Taxonomic classification of mouse gut metagenomes

Filtered paired-end metagenomic reads were used for taxonomic profiling of mouse gut metagenomes with Kraken v2.1.2 and Bracken v2.6.2 using the Mouse Gastrointestinal Bacteria Catalogue (MGBC) database^17^, as described above. To predict the LPS-producing bacterial species in the mouse metagenomes, *lpx* operon genes were identified using the MGBC protein database^17^. The abundance data were normalized by the weight of the faecal pellet used for the DNA extraction. Data were analysed and visualized in R.

### Mass spectrometry of acylation of lipid A from commensal gut bacteria

Mass spectrometry analyses of lipid A were performed using an Agilent 1290 Infinity II LC system coupled to an Agilent 6546 Q-TOF instrument. Lipid A-1P standard (Sigma-Aldrich catalogue number L6638-1MG) was prepared in chloroform/methanol/water (74:23:3) at 1 mg ml^−1^. About 5 µl of 0.5 mg ml^−1^ of lipid A-1P was injected onto a Waters XBridge C18 column (50 mm × 2.1 mm, 3.5 µm particle size), and the mobile phase was a linear gradient of 0–95% isopropyl alcohol with 200 mM ammonium hydroxide and 5 µM medronic acid (mobile phase B) in methanol/water (80:20) with 200 mM ammonium hydroxide and 5 µM medronic acid (mobile phase A) over 17 min (before holding at 100% mobile phase A for 3 min). The flow rate was 0.25 ml min^−1^, and the column was operated at 45 °C. Post-column addition of 10% DMSO in acetone at a rate of 0.25 ml min^−1^ was used. The published extracted ion chromatograms by Sándor et al.^52^ were used to characterize the lipid A molecule. Increases in acyl chain length led to later chromatographic elution.

Mouse commensal *Escherichia coli* (isolate A7_7.EC.CDM; NCBI assembly GCA_910574425.1) and *Klebsiella pneumoniae* (isolate A5_5.KP.CDM; NCBI assembly GCA_910574035.1) from our mouse culture collection were confirmed to encode *lpxM* using the MGBC ToolKit with ‘feature_search’ function. These isolates were inoculated in 5 ml of yeast casitone fatty acid (YCFA) broth using a single colony from streaked YCFA agar plates. The cultures were incubated anaerobically overnight at 37 °C. The 5 ml cultures were used to inoculate 1 l of YCFA liquid media. One-litre liquid cultures were incubated anaerobically overnight at 37 °C to an optical density at 600 nm of approximately 1.000. Cultures were separated into 200 ml tubes and centrifuged at 3,500 × *g* for 20 min. Media was discarded, and bacterial cell pellets were washed once with PBS. Dry pellets were stored at −80 °C. The culture pellet was prepared at 100 mg ml^−1^ in water, hydrolysed with a mild acid hydrolysis buffer (50 mM sodium acetate), and Bligh-Dyer extracted (proportion of chloroform/methanol/water, 1:2:0.8). Final resuspension was in chloroform/methanol/water (74:23:3), and 5 µl was injected onto the Waters XBridge C18 column (50 mm × 2.1 mm, 3.5 µm particle size), using the same chromatographic conditions as described above. The acyl chain lengths for the monophosphorylated and diphosphorylated forms of lipid A were annotated based on Sándor et al.^52^, and the proportional composition of these acyl chains was calculated for each isolate.

### Tumour challenge and treatment

MC38 colon carcinoma cells were purchased from Kerafast. Cells were passaged in DMEM (Sigma-Aldrich, D5796) supplemented with 10% fetal bovine serum (FBS) (Sigma-Aldrich BCCC3714), 1% Glutamax (Thermo Fisher Scientific, 35050-038), 1% non-essential amino acids (Thermo Fisher Scientific, 11140-035), 1% sodium pyruvate (Thermo Fisher Scientific, 11360-039), 1% penicillin–streptomycin (Thermo Fisher Scientific, 15140-122), 0.1% of 2-mercaptoethanol (Thermo Fisher Scientific, 21985023), amphotericin B (Thermo Fisher Scientific, 15290-026) and gentamycin (Thermo Fisher Scientific, 15750-045). Mice under isoflurane anaesthesia were injected subcutaneously with 10^6^ cells in 100 µl of sterile 1× PBS in the right flank. Tumours were measured using callipers at days 7, 10, 14, 17 and 20 after implantation. To control for incomplete tumour engraftment, mice with a tumour volume of less than 27 mm^3^ at day 7 after implantation were removed from the study. Mice were injected intraperitoneally (i.p.) with 200 µg anti-PD-1 (clone 29F.1A12) (BioXCell, BE0273) or IgG2a isotype control (BioXcell, BE0089) twice a week. Hexa- and penta-acylated LPS were purchased from Sigma-Aldrich (L2630-100MG and L9143-100MG). Mice were administered LPS (25 mg l^−1^) in sterile drinking water starting 10 days after tumour implantation. In line with our animal ethics guidelines, mice were culled if their tumour reached 1.5 cm in diameter or if the tumour became ulcerated. LPS-containing water was changed twice a week. The TLR4 inhibitor Resatorvid (TAK-242) (APExBIO, A3850) was administered i.p. on days 10, 12, 14, 17 and 19 post tumour implantation (60 μg per injection).

### Sample processing of murine tumours, spleens, mesenteric lymph nodes and tumour-draining lymph nodes for immunophenotyping

Tumour and spleen samples were digested in Roswell Park Memorial Institute (RPMI) medium containing collagenase D and DNase I (Sigma-Aldrich, 11088882001 and 10104159001) at 37 °C for 30 min before dissociation through 70 µm cell strainers. A 40/80 Percoll gradient was used to isolate lymphocytes from tumours, and resulting cell suspensions were filtered using 40 µm cell strainers. For spleen samples, Red Blood Cell Lysis Buffer (Sigma-Aldrich, R7757-100ML) was applied at room temperature for 5 min to selectively lyse red blood cells. Mesenteric lymph nodes and tumour-draining lymph nodes were digested in complete HBSS with 2% FBS containing collagenase D at 37 °C for 30 min and dissociated through 70 µm cell strainers.

### Flow cytometry of tumour-infiltrating and lymphoid tissue lymphocytes

Isolated lymphocytes were re-stimulated with 1 µg ml^−1^ Brefeldin A (eBioscience, 00-4506-51), 50 ng ml^−1^ phorbol 12-myristate 13-acetate (Sigma-Aldrich, P8139-1MG) and 1 µg ml^−1^ ionomycin (Sigma-Aldrich, I0634-1MG) in complete RPMI with 10% FBS in a 37 °C cell culture incubator with 5% CO_2_ for 4.5 h. Dead cells were then stained using the LIVE/DEAD Fixable Aqua Dead Cell Stain Kit (Thermo Fisher Scientific, L34965). Cells were then stained with extracellular antibodies for 20 min on ice and permeabilized for 30 min using the BD Cytofix/Cytoperm Fixation/Permeabilization Kit (BD Biosciences, 554714) according to the manufacturer’s protocol. The intracellular antibodies listed in Supplementary Table 6 were then added and incubated for 30 min on ice. Cells were post-fixed in 1% PFA at 4 °C until analysis on a Cytek Aurora flow cytometer. Samples with cell viability below 30% were excluded from further analyses. Quantification of cell numbers was achieved using CountBright Absolute Counting Beads (Thermo Fisher Scientific, C36950), in accordance with the protocol provided by the manufacturer. Data were exported as FCS files using Cytek Aurora software (v3.3.0) and analysed using FlowJo software (v10.8.1, Tree Star). For lymphocyte flow cytometry antibody panel, see Supplementary Table 6.

### Flow cytometry of myeloid cells

The TruStain FcX Antibody (BioLegend, 101320) was used to block cells for 5 min at 4 °C. Dead cells were then stained using the LIVE/DEAD Fixable Aqua Dead Cell Stain Kit. After washing, cells were stained with the surface antibodies listed in Supplementary Table 6 in 1× PBS for 20 min on ice. Cells were fixed in 1% PFA at 4 °C until analysis on a Cytek Aurora flow cytometer. Data were exported as FCS files using Cytek Aurora software (v3.3.0) and analysed using FlowJo software (v10.8.1, Tree Star). The myeloid flow cytometry antibody panel is shown in Supplementary Table 6.

### In vitro NF-kB activation in monocyte/macrophages

THP-1 cells (THP1-Dual Reporter Cells, TLR2 KO Dual Reporter THP-1 Cells, TLR4 KO Dual Reporter THP-1 Cells and THP1-Dual MD2-CD14-TLR4 over-expressing THP-1 cells) were purchased from InvivoGen. Cells were cultured in RPMI 1640 (Gibco) with 2 mM l-glutamine (Sigma-Aldrich), 25 mM HEPES (Gibco), 10% FBS (Sigma-Aldrich), 100 µg ml^−1^ Normocine (InvivoGen), 100 U ml^−1^ penicillin–streptomycin (Gibco), 10 µg ml^−1^ blasticidin (InvivoGen) and 100 µg ml^−1^ Zeocin (InvivoGen) according to the manufacturer’s instructions. For NF-kB activation assays, THP-1 cells were resuspended in Test medium (RPMI 1640 with 2 mM l-glutamine, 25 mM HEPES, 10% FBS and 100 U ml^−1^ penicillin–streptomycin) and cultured at 10^5^ cells per well for 24 h in a final volume of 200 µl of Test medium with indicated concentrations of ultrapure LPS from *E. coli* (hexa LPS, InvivoGen) and/or ultrapure LPS from *Porphyromonas* *gingivalis* (penta LPS, InvivoGen) with or without PMB (APExBio) in CELLSTAR 96-well plates (Greiner). About 20 µl^−1^ of culture supernatants was mixed with 180 µl of QUANTI-Blue Solution (InvivoGen) in 96-well enzyme-linked immunosorbent assay microplates (Greiner) according to the manufacturer’s instructions. The plates were incubated at 37 °C for 1–6 h. Optical density at 630 nm was measured on a FLUOstar Omega (BMG Labtech).

### In vitro cytokine secretion in monocyte/macrophages

Human THP-1 cells were cultured as indicated above, and supernatants were collected at 24 h. The indicated cytokine cytometric beads and paired-end-conjugated detection antibodies from the BD Cytometric Bead Array Human Inflammatory Cytokines Kit (BD Bioscience) were mixed with these supernatants according to the manufacturer’s instructions. Mouse RAW 264.7 cells (Merck, catalogue number 91062702-1VL) were cultured in high-glucose DMEM (Sigma-Aldrich) with 10% FBS (Sigma-Aldrich) and 100 U ml^−1^ penicillin–streptomycin (Gibco). About 10^5^ cells per well were cultured for 24 h in a final volume of 200 µl culture medium with indicated concentrations of ultrapure LPS from *E. coli* (hexa LPS, InvivoGen) and/or ultrapure LPS from *P. gingivalis* (penta LPS, InvivoGen) with or without PMB (APExBio) in CELLSTAR 96-well plates (Greiner). Culture supernatants were mixed with the indicated cytokine beads and paired-end-conjugated detection antibodies from the Mouse Th1/Th2/Th17 CBA Kit (BD Bioscience) according to the manufacturer’s instructions. All samples were analysed on a BD LSRFortessa Cell Analyzer (BD Bioscience).

### Statistical analysis

Statistical analyses were performed using either Graphpad Prism software (v9.3.1) or R (v4.3.1). Non-parametric Kruskal–Wallis tests and parametric Brown–Forsythe tests were used as indicated to calculate statistical significance of the difference in sample medians and means, respectively, and post hoc Dunn *P* values with Bonferroni false discovery rate adjustment were calculated for the pairwise comparisons among the enterotypes. The proportions of clinical responders were compared between enterotypes using one-tailed two proportions *Z*-tests without continuity correction. Two-way analysis of variance (ANOVA) was used to evaluate statistical significance in experiments with tumour growth measurements. We used NMDS, a distance-based ordination technique^53,54^ in R (vegan::metaMDS()) with default engine = “monoMDS”. In the process of calculation of goodness of fit, a single NMDS run identifies a local minimum; therefore, we iterated the runs 1,000 times, or the best solution achieved to reach the global minimum (convergence) using the function Procrustes in R (vegan:: procrustes()). The final stress score was used to assess the optimal model and goodness of fit as suggested previously^55,56^. In addition to the stress score, we also performed a two-tailed *t*-test on the NMDS coordinates as shown previously^57^ to highlight the difference between the ‘Responder’ and ‘Non-responder’ groups. *P* values of less than 0.05 were considered statistically significant. Statistical tests used are specified in the figure legends. Data distribution was assumed to be normal/parametric or non-parametric based on the data type, but this was not formally tested. *P* values correlate with symbols as follows: NS, not significant; *P ≤ 0.05; **P ≤ 0.01; ***P ≤ 0.001; ****P ≤ 0.0001. No statistical methods were used to pre-determine sample sizes, but where relevant, sample sizes were determined based on variability observed in published experiments of a similar kind^2^. In some experiments prior experience of sample size requirement was used to design experimental group sizes. For experiments where technical limitations prevented adequate statistical power to be obtained from single experiments, results from multiple experiments were pooled to provide sufficient statistical power. Data reported are in most cases non-subjective and did not require randomization or blinding at measurement. Where possible, data collection and/or the organization of the experimental conditions were randomized. Investigators were not formally blinded. However, in randomized experiments it was difficult for investigators and technicians to readily determine treatment groups from animal IDs at the bench.

### Reporting summary

Further information on research design is available in the Nature Portfolio Reporting Summary linked to this article.

## Supplementary information

Reporting Summary

Supplementary Tables Supplementary Tables 1–6.

## Source data

Source Data Fig. 1 Statistical source data.

Source Data Fig. 2 Statistical source data.

Source Data Fig. 3 Statistical source data.

Source Data Fig. 4 Statistical source data.

Source Data Extended Data Fig. 2 Statistical source data.

Source Data Extended Data Fig. 3 Description and accession information for mass spectrometry data.

Source Data Extended Data Fig. 4 Statistical source data.

Source Data Extended Data Fig. 6 Statistical source data.

Source Data Extended Data Fig. 7 Statistical source data.

Source Data Extended Data Fig. 8 Statistical source data.

## Data Availability

Mouse faecal metagenomic sequences generated in this study are deposited in the NCBI SRA under Bioproject ID PRJNA1171992. Metagenomes of patients undergoing immunotherapy treatments were previously published and deposited in the NCBI SRA under the accession numbers ERP127050, SRP339782, SRP197281, SRP116709, SRP115355 and ERP104577. Taxonomic profiling was performed using a custom database built from representative genomes of the human gut microbiota that were re-annotated using the Genome Taxonomy Database v2.1. This database is available via Zenodo at 10.5281/zenodo.7319344 (ref. ^48^). Human and mouse genomes GRCh38 and GRCm39, respectively, were used to remove host contamination from the shotgun metagenomics sequencing reads. LPS mass spectrometry files have been deposited in MassIVE under accession number MSV000096803. This project can be accessed at ftp://massive.ucsd.edu/v06/MSV000096803/. Source data are provided with this paper.

## Code availability

This paper did not generate new code.
