# Supplementary material for: Gut microbiota-derived hexa-acylated lipopolysaccharides enhance cancer immunotherapy responses
Source: Nat Microbiol. 2025 Feb 10;10(3):795–807. doi: 10.1038/s41564-025-01930-y (PMC11879847; doi:10.1038/s41564-025-01930-y)
Supplement: Supplementary file 1 — Reporting Summary [file 41564_2025_1930_MOESM1_ESM.pdf]

nature portfolio

Corresponding author(s): Virginia A. Pedicord
Last updated by author(s): Jan 8, 2025

Reporting Summary

Nature Portfolio wishes to improve the reproducibility of the work that we publish. This form provides structure for consistency and transparency in reporting. For further information on Nature Portfolio policies, see our Editorial Policies and the Editorial Policy Checklist.

Statistics

For all statistical analyses, confirm that the following items are present in the figure legend, table legend, main text, or Methods section.

n/a	Confirmed	
☐	☒	The exact sample size (*n*) for each experimental group/condition, given as a discrete number and unit of measurement
☐	☒	A statement on whether measurements were taken from distinct samples or whether the same sample was measured repeatedly
☐	☒	The statistical test(s) used AND whether they are one- or two-sided<br>*Only common tests should be described solely by name; describe more complex techniques in the Methods section.*
☒	☐	A description of all covariates tested
☐	☒	A description of any assumptions or corrections, such as tests of normality and adjustment for multiple comparisons
☐	☒	A full description of the statistical parameters including central tendency (e.g. means) or other basic estimates (e.g. regression coefficient) AND variation (e.g. standard deviation) or associated estimates of uncertainty (e.g. confidence intervals)
☐	☒	For null hypothesis testing, the test statistic (e.g. *F*, *t*, *r*) with confidence intervals, effect sizes, degrees of freedom and *P* value noted<br>*Give P values as exact values whenever suitable.*
☒	☐	For Bayesian analysis, information on the choice of priors and Markov chain Monte Carlo settings
☒	☐	For hierarchical and complex designs, identification of the appropriate level for tests and full reporting of outcomes
☒	☐	Estimates of effect sizes (e.g. Cohen's *d*, Pearson's *r*), indicating how they were calculated

Our web collection on statistics for biologists contains articles on many of the points above.

Software and code

Policy information about availability of computer code

Data collection	Raw uncompensated flow cytometry was acquired on a Cytek Aurora instrument and exported as FCS files using Cytek Aurora software (v3.3.0). Data were compensated and analysed as described below.<br><br>Sequence data:<br>National Center for Biotechnology Information (NCBI); NCBI sra-tools (v2.11.2)
Data analysis	Raw uncompensated flow cytometry data, exported as FCS files, were compensated using compensation controls during each acquisition, and analysed using FlowJo software (v10.8.1, Tree Star, Inc.).<br><br>Statistics: Graphpad Prism software (v9.3.1), R (v4.3.1)<br><br>Bioinformatics:<br>FastQC (v0.11.9); multiqc (v1.11); Trimmomatic (v0.39); Bowtie 2 (v2.3.5); Kraken (v2.1.2); Bracken (v2.6.2); MGBC-Toolkit v1.0; metaSPAdes (v3.15.4); MEGAHIT (v1.2.9); CD-HIT (v4.8.1); Prodigal (v2.6.3); Diamond (v2.0.15.153); SqueezeMeta (v1.5.2); Salmon tool (v1.8.0); R-packages : ComBat-Seq, ConQuR (v1.2.0), clusterSim, ade4, and vegan

For manuscripts utilizing custom algorithms or software that are central to the research but not yet described in published literature, software must be made available to editors and reviewers. We strongly encourage code deposition in a community repository (e.g. GitHub). See the Nature Portfolio guidelines for submitting code & software for further information.

Data

Policy information about availability of data

All manuscripts must include a data availability statement. This statement should provide the following information, where applicable:

- Accession codes, unique identifiers, or web links for publicly available datasets
- A description of any restrictions on data availability
- For clinical datasets or third party data, please ensure that the statement adheres to our policy

Mouse fecal metagenomic sequences generated in this study are deposited in the NCBI Sequence Read Archive (SRA) under Bioproject ID: PRJNA1171992. Immunotherapy patient metagenomes were previously published and deposited in the NCBI SRA under the accession numbers ERP127050, SRP339782, SRP197281, SRP116709, SRP115355 and ERP104577. Taxonomic profiling was performed using a custom database built from representative genomes of the human gut microbiota that were re-annotated using the Genome Taxonomy Database (GTDB) v2.1. This database is available at doi.org/10.5281/zenodo.7319344. Human and mouse genomes GRCh38 and GRCm39, respectively, were used to remove host contamination from the shotgun metagenomics sequencing reads. LPS mass spectrometry files have been deposited in MassIVE under accession number MSV000096803. This project can be accessed at: ftp://massive.ucsd.edu/v06/MSV000096803/.

Research involving human participants, their data, or biological material

Policy information about studies with human participants or human data. See also policy information about sex, gender (identity/presentation), and sexual orientation and race, ethnicity and racism.

Reporting on sex and gender	N/A
Reporting on race, ethnicity, or other socially relevant groupings	N/A
Population characteristics	N/A
Recruitment	N/A
Ethics oversight	N/A

Note that full information on the approval of the study protocol must also be provided in the manuscript.

Field-specific reporting

Please select the one below that is the best fit for your research. If you are not sure, read the appropriate sections before making your selection.

☒ Life sciences ☐ Behavioural & social sciences ☐ Ecological, evolutionary & environmental sciences

For a reference copy of the document with all sections, see nature.com/documents/nr-reporting-summary-flat.pdf

Life sciences study design

All studies must disclose on these points even when the disclosure is negative.

Sample size	Where relevant, sample sizes were determined based on variability observed in published experiments of a similar kind (DOI: 10.1126/science.aan3706). In some experiments prior experience of sample size requirement was used to design experimental group sizes. For experiments where technical limitations prevented adequate statistical power to be obtained from single experiments, results from multiple experiments were pooled to provide sufficient statistical power.
Data exclusions	Flow cytometry samples which had undergone technical failure during processing or which had insufficient numbers of viable cells were excluded from analyses. Pre-established exclusion criteria across samples from a given experiment were used to avoid subjective bias. Experiments included positive controls to allow technical failure to be objectively determined.
Replication	The number of independently repeated experiments for each finding are described in the figure legends. All technically successful replicate experiments reproduced the indicated findings.
Randomization	Data reported are in most cases non-subjective and did not require randomization or blinding at measurement.
Blinding	Investigators were not formally blinded. However, in randomized experiments it was difficult for investigators and technicians to readily determine treatment groups from animal IDs at the bench.

Reporting for specific materials, systems and methods

We require information from authors about some types of materials, experimental systems and methods used in many studies. Here, indicate whether each material, system or method listed is relevant to your study. If you are not sure if a list item applies to your research, read the appropriate section before selecting a response.

Materials & experimental systems

n/a	Involved in the study	
☐	☒	Antibodies
☐	☒	Eukaryotic cell lines
☒	☐	Palaeontology and archaeology
☐	☒	Animals and other organisms
☐	☒	Clinical data
☒	☐	Dual use research of concern
☒	☐	Plants

Methods

n/a	Involved in the study	
☒	☐	ChIP-seq
☐	☒	Flow cytometry
☒	☐	MRI-based neuroimaging

Antibodies

Antibodies used	anti-CD45, BUV805, (clone 30-F11), Thermo Cat# 368-0451-80<br>anti-TCRβ, APC-eFluor 780, (clone H57-597), Thermo Cat# 47-5961-82<br>anti-TCRγ, FITC, (clone GL-3), Thermo Cat# 11-5711-82<br>anti-CD4, PE-Cy7, (clone RM4-5), Thermo Cat# 25-0042-82<br>anti-CD8α, BUV737, (clone 53-6.7), Thermo Cat# 367-0081-82<br>anti-IFNγ, Percp-Cy5.5, (clone XMG1.2), Thermo Cat# 45-7311-82<br>anti-TNFα, APC, (clone MP6-XT22), Thermo Cat# 17-7321-82<br>anti-Foxp3, eFluor450, (clone FJK-16s), Thermo Cat# 48-5773-82<br>anti-Ki-67, BUV 395, (clone SolA15), Thermo Cat# 363-5698-82<br>anti-CD103, PerCP-Cy5.5, (clone 2E7), BioLegend Cat# 121415<br>anti-CD5, FITC (DUMP), (clone 53-7.3), Thermo Cat# 11-0051-82<br>anti-CD19, FITC (DUMP), (clone eBio1D3 (1D3)), Thermo Cat# 363-0193-82<br>anti-CD3e, FITC (DUMP), (clone 145-2C11), Thermo Cat# 11-0031-85<br>anti-B220, FITC (DUMP), (clone RA3-6B2), Thermo Cat# 11-0452-82<br>anti-CD45, Super Bright 600, (clone 30-F11), Thermo Cat# 63-0451-82<br>anti-F4/80, eFluor 450, (clone BM8), Thermo Cat# 48-4801-82<br>anti-CD11b, Brilliant Violet 786, (clone M1/70), Thermo Cat# 417-0112-82<br>anti-MHC II (I-A/I-E) , Spark UV 387, (clone M5/114.15.2), BioLegend Cat# 107670<br>anti-CD64, APC, (clone X54-5/7.1), BioLegend Cat# 139306<br>anti-CD11c, Alexa Fluor 700, (clone N418), Thermo Cat# 56-0114-82<br>anti-CD86, APC-Cy7, (clone GL-1), BioLegend Cat# 105029<br>anti-CD206, PE, (clone C068C2), BioLegend Cat# 141705<br>anti-PD1 (clone 29F.1A12), BioXcell Cat# BE0273-25MG<br>rat IgG2a isotype control (clone 2A3), BioXcell Cat# BE0089-25MG<br>TruStain FcX Antibody (BioLegend Cat# 141705 101320)
Validation	Antibody validation information is available for each of the listed antibodies on the relevant manufacturer websites.

Eukaryotic cell lines

Policy information about cell lines and Sex and Gender in Research

Cell line source(s)	MC38 colon carcinoma cells were purchased from Kerafast.<br>THP-1 cells (THP1-Dual Reporter Cells, TLR2 KO Dual Reporter THP-1 Cells, TLR4 KO Dual Reporter THP-1 Cells and THP1-Dual MD2-CD14-TLR4 over-expressing THP-1 cells) were purchased from InvivoGen.<br>RAW 264.7 was obtained from Merck, catalog# 91062702-1VL.
Authentication	No cell line authentication was performed; Low passage stocks were used
Mycoplasma contamination	Cell lines were screened for mycoplasma and found to be negative prior to use.
Commonly misidentified lines<br>(See ICLAC register)	No commonly misidentified cell lines were used.

Animals and other research organisms

Policy information about studies involving animals; ARRIVE guidelines recommended for reporting animal research, and Sex and Gender in Research

Laboratory animals	9-week-old C57BL/6 mice purchased from Charles River.
Wild animals	Wild animals were not used in this study.

Reporting on sex | *Indicate if findings apply to only one sex; describe whether sex was considered in study design, methods used for assigning sex. Provide data disaggregated for sex where this information has been collected in the source data as appropriate; provide overall numbers in this Reporting Summary. Please state if this information has not been collected. Report sex-based analyses where performed, justify reasons for lack of sex-based analysis.*

Field-collected samples | The study did not involve samples collected from the field.

Ethics oversight | Experiments were conducted in accordance with UK Home Office guidelines and were approved by the University of Cambridge Animal Welfare and Ethical Review Board.

Note that full information on the approval of the study protocol must also be provided in the manuscript.

Clinical data

Policy information about clinical studies

All manuscripts should comply with the ICMJE guidelines for publication of clinical research and a completed CONSORT checklist must be included with all submissions.

Clinical trial registration | *Provide the trial registration number from ClinicalTrials.gov or an equivalent agency.*

Study protocol | *Note where the full trial protocol can be accessed OR if not available, explain why.*

Data collection | *Describe the settings and locales of data collection, noting the time periods of recruitment and data collection.*

Outcomes | *Describe how you pre-defined primary and secondary outcome measures and how you assessed these measures.*

Plants

Seed stocks | *Report on the source of all seed stocks or other plant material used. If applicable, state the seed stock centre and catalogue number. If plant specimens were collected from the field, describe the collection location, date and sampling procedures.*

Novel plant genotypes | *Describe the methods by which all novel plant genotypes were produced. This includes those generated by transgenic approaches, gene editing, chemical/radiation-based mutagenesis and hybridization. For transgenic lines, describe the transformation method, the number of independent lines analyzed and the generation upon which experiments were performed. For gene-edited lines, describe the editor used, the endogenous sequence targeted for editing, the targeting guide RNA sequence (if applicable) and how the editor was applied.*

Authentication | *Describe any authentication procedures for each seed stock used or novel genotype generated. Describe any experiments used to assess the effect of a mutation and, where applicable, how potential secondary effects (e.g. second site T-DNA insertions, mosiacism, off-target gene editing) were examined.*

Flow Cytometry

Plots

Confirm that:

☒ The axis labels state the marker and fluorochrome used (e.g. CD4-FITC).

☒ The axis scales are clearly visible. Include numbers along axes only for bottom left plot of group (a 'group' is an analysis of identical markers).

☒ All plots are contour plots with outliers or pseudocolor plots.

☒ A numerical value for number of cells or percentage (with statistics) is provided.

Methodology

Sample preparation | As described in the manuscript, tumor and spleen samples were digested in RPMI containing collagenase D and DNase I (Sigma-Aldrich, 11088882001 and 10104159001) at 37°C for 30 minutes before dissociation through 70-µm cell strainers. A 40/80 Percoll gradient was used to isolate lymphocytes from tumors, and resulting cell suspensions were filtered using 40-µm cell strainers. For spleen samples, Red Blood Cell Lysis Buffer (Sigma-Aldrich, R7757-100ML) was applied at room temperature for 5 minutes to selectively lyse red blood cells. Mesenteric lymph nodes (mLNs) and tumor draining lymph nodes (TDLNs) were digested in complete HBSS with 2% FBS containing collagenase D at 37°C for 30 minutes and dissociated through 70-µm cell strainers. Isolated lymphocytes were re-stimulated with 1 µg/mL Brefeldin A (eBioscience™, 00-4506-51), 50 ng/mL Phorbol 12-myristate 13-acetate (PMA) (Sigma-Aldrich, P8139-1MG), and 1 µg/mL ionomycin (Sigma-Aldrich, I0634-1MG) in complete RPMI with 10% FBS in a 37°C cell culture incubator with 5% CO2 for 4.5 hours. Dead cells were then stained using the LIVE/DEAD™ Fixable Aqua Dead Cell Stain Kit (Thermo Fisher Scientific, L34965). Cells were then stained with extracellular antibodies (table below) for 20 minutes on ice and permeabilized for 30 minutes using the BD Cytofix/Cytoperm™ Fixation/Permeabilization Kit (BD Biosciences, 554714) according to the manufacturer's protocol. The following intracellular antibodies were then added and incubated for 30 minutes on ice. Cells were post-fixed in 1% PFA at 4°C until analysis on a Cytek® Aurora flow cytometer.

Instrument	Samples were analysed using an Aurora Cytek instrument.
Software	Data were analyzed using FlowJo™ software (v10.8.1, Tree Star, Inc.)
Cell population abundance	The analysis focused on tumor-infiltrating and peripheral immune cells, including cytokine-secreting T cells, dendritic cells (DCs), and macrophages. The proportions of these cell populations were determined, with detailed frequencies available in the result figures and extended data figures. Counting beads were utilized to provide an accurate cell number count, ensuring reliable quantification of each cell type. This comprehensive approach highlights the dynamics of immune cell populations within the tumor microenvironment and their potential role in the immune response.
Gating strategy	In general, cells were gated based on a viability gate using amine-reactive viability exclusion dye intensity, followed by a lymphocyte gate, a singlet gate, and then subsequent gating based on the expression of surface and intracellular proteins as described in the manuscript. This is also shown in the extended data figures.

☒ Tick this box to confirm that a figure exemplifying the gating strategy is provided in the Supplementary Information.
